# Portable Oxygen Therapy: Is the 6-Minute Walking Test Overestimating the Actual Oxygen Needs?

**DOI:** 10.3390/jcm9124007

**Published:** 2020-12-11

**Authors:** Daniel Sanchez-Morillo, Alejandro Lara-Doña, Blanca Priego-Torres, Maria Morales-Gonzalez, Francisca Montoro-Ballesteros, Antonio Leon-Jimenez

**Affiliations:** 1 Biomedical Research and Innovation Institute of Cadiz (INiBICA), Hospital Universitario Puerta del Mar, Avda. Ana de Viya 21, 11009 Cádiz, Spain; alejandro.lara@uca.es (A.L.-D.); blanca.priego@uca.es (B.P.-T.); antonio.leon.sspa@juntadeandalucia.es (A.L.-J.); 2 Biomedical Engineering and Telemedicine Research Group, Department of Automation Engineering, Electronics and Computer Architecture and Networks, School of Engineering, University of Cadiz, Avda. Universidad de Cádiz 10, Puerto Real, 11519 Cádiz, Spain; 3 Pulmonology, Allergy and Thoracic Surgery Unit, Puerta del Mar University Hospital, Avda. Ana de Viya 21, 11009 Cádiz, Spain; moralesgonzalez.maria@gmail.com (M.M.-G.); paquimb_88@hotmail.com (F.M.-B.)

**Keywords:** COPD, oxygen therapy, 6-minute walk test, CT90, oximetry, oxygen therapy

## Abstract

The appropriate titration for the personalized oxygen needs of patients with chronic obstructive pulmonary disease (COPD) and severe hypoxemia is a determining factor in the success of long-term oxygen therapy. There are no standardized procedures to assist in determining the patient’s needs during the physical activities of daily life. Despite that effort tests are a wide broad approach, further research concerning the development of protocols to titrate O_2_ therapy is needed. The main objective of this study was to assess whether the level of oxygen titrated through the 6-minute walking test (6MWT) for patients with COPD and exertional hypoxemia is adequate to meet the patients’ demand during their activities of daily living. Physiological and subjective variables were estimated for a study population during two walking tests: a 6MWT and a 20-minute walking circuit (20MWC), designed ad-hoc to reproduce daily physical activities more truthfully. The results indicate that in a significant proportion of patients, the 6MWT might not accurately predict their oxygen needs at a domiciliary environment. Therefore, the titration of the portable O_2_ therapy could not be optimal in these cases, with the detrimental impact on the patient’s health (hyperoxia episodes), the autonomy of the oxygen device, and the decrease of time out of the home.

## 1. Introduction

Since the studies carried out in the 1980s in which both the Nocturnal Oxygen Therapy Trial [1] and the British Medical Research Council study [2] demonstrated the benefits of oxygen therapy on the survival of patients with chronic obstructive pulmonary disease (COPD) and severe hypoxemia, the use of this therapy has not ceased to grow, multiplying its incidence by more than three times in some countries [3] and with an estimated global cost of 18.2 billion by 2024 [4].

In the general context of COPD, long-term supplemental oxygen therapy (LTOT) is particularly relevant for a variable percentage of patients. It has been reported that 47% of patients with COPD experience episodes of desaturation during exercise that manifest themselves with SpO_2_ values < 88% in effort tests and during the practice of activities of daily living [5,6,7].

In these cases, the indication for LTOT during exercise aims to prevent hypoxemia by raising O_2_ saturation levels above 90%, improving tissue oxygenation, delaying the onset of hyperinflation and associated dyspnoea, and increasing exercise capacity in subjects with moderate and severe COPD [8]. In other respiratory processes such as pulmonary fibrosis, exercise oxygen therapy has been shown to improve the quality of life of these patients [9].

However, beyond the reported benefits of oxygen therapy, the associated technology can be challenging for physicians, caregivers, providers and patients [10], especially in the processes of prescription, titration and selection and management of delivery devices in a scenario complicated by national regulations [11].

Portable devices for the oxygen supply during activities carried out outside the home and the appropriate titration for the personalized needs of the patients are determining factors in the success of LTOT. Although there may be different criteria in national and international guidelines for its indication [12], they all agree on the need to carry out some kind of effort or stress test for the diagnosis of hypoxemia at exercise and the titration of the oxygen needed for its correction to assure the correct supply of oxygen during life activities.

Notwithstanding the progress made in the development of a new generation of devices for the automatic adjustment of oxygen flow to the changing needs of individual patients, these closed-loop O_2_ supply systems have not yet been incorporated into routine clinical practice [13], and therapy titration remains a manual procedure. There are no standardized procedures to assist in determining the patient’s needs during the physical activities of daily life. The most widespread approach is to sequentially employ multiple effort tests by progressively increasing O_2_ supplementation until the percentage of oxygen bound to haemoglobin in the blood remains above 88% or 90% during the test. This is a time-consuming methodology that is often difficult to complete for patients with chronic lung diseases [14].

Timed walking tests are good predictors of survival [15] and are the most frequently used to assess exercise capacity, prognosis, and response to treatment in a wide range of respiratory diseases [16,17,18]. Among them, the most prevalent is the 6-minute walking test (6MWT), which is recommended by some national regulations to this purpose [16,19,20,21].

The use in clinical trials of walking tests such as the 6MWT instead of cycling or treadmill tests is partly based on the fact that it is a more natural form of exercise that can better represent daily activities allowing a better assessment of the physical, psychological and emotional capabilities of patients [17].

The 6MWT is a sub-maximal test of high intensity and constant load. It is a self-regulating test in which patients determine their walking speed, even though they are instructed to walk as far as possible, walking as fast as they can, without running or jogging. In this test, patients often start with a high walking pace to progressively reach a constant speed at which they feel comfortable [22].

Nonetheless, a submaximal and constant speed is not the usual behaviour during the activities of the daily life of a patient with respiratory disease and home oxygen therapy. Very recent studies conducted to describe the physical activity performed in their usual environment by chronic respiratory patients have reported high rates of sedentarism. Moderate to intense physical activity presents metabolic equivalents of tasks (METs) greater than three. However, the vast majority of daily activities in daily life carried out by patients with COPD can be considered as light (1.5 < METs < 3) or sedentary (METs < 1.5) [23]. These patients carry out activities with an energy expenditure fewer than three METs for 99.6% of the daily time [24] and spend more than 40% with sedentary behaviour [23,24].

Patients receiving LTOT usually take walks at non-submaximal speed as one of the most common activities, and therefore titration at a higher load level than really necessary may overestimate the need for O_2_, increasing the risk of hyperoxia, whose harmful effects are well known and often minimized by healthcare professionals [13]. The exposure of patients with COPD to prolonged periods of hyperoxemia has been linked to higher mortality and worsening clinical outcomes [25,26]. Additionally, the supply of an excessive oxygen flow induces unnecessarily high oxygen consumption, a decrease in the battery life of portable devices or oxygen cylinders and consequently a limitation in the autonomy of the patient.

Considering the existing need for further research concerning the development of protocols to assess O_2_ therapy [27,28], the main objective of this study is to assess whether the level of oxygen titrated through the 6MWT for patients with chronic respiratory insufficiency and exertional hypoxemia is adequate to meet the demand caused in the patient by their activities of daily living. These routine activities were reproduced in a hospital environment by a 20-minute walking circuit (20MWC). The primary aim was to contribute to the optimization of therapy and patient safety.

## 2. Methods

### 2.1. Participants

In the absence of data to estimate the sample size at the start of this study, 17 patients from the University Hospital Puerta del Mar in Cadiz (UHPM) were arbitrarily enrolled in this exploratory study, with the hypothesis that this number would be sufficient to detect significant variability in the SpO_2_ ranges. The inclusion criteria addressed patients with chronic respiratory insufficiency or exertional hypoxemia, treated with portable oxygen therapy, and who required a certain flow of O_2_ at rest and a different one on exertion. A minimum experience of at least three months using a portable oxygen therapy device was considered necessary.

All subjects had at least one titration at rest and one through a 6MWT. The O_2_ dosage at rest was being verified every three months and the dose at effort was being updated at least once a year. The goal in these titration procedures was to maintain a SpO_2_ level between 90% and 92% during the 6MWT.

The selected subjects were routinely engaged in at least two hours of daily activity wandering outside the home. All patients were clinically stable with no symptoms of exacerbation of their respiratory disease for at least six weeks before this pilot and had no significant comorbidities.

Exclusion criteria involved: (a) any significant organic co-morbidity that could cause or contribute to exertional dyspnoea hindering the completion of the circuit (cardiovascular, metabolic or other associated respiratory diseases); (b) active smoking; (c) exacerbation of COPD in the six weeks before acceptance for participation in the study, or any disease that could limit the patient’s physical activity (e.g., neuromuscular or skeletal diseases); and (d) general frailty (i.e., difficulty in walking or lack of autonomy) that could substantially prevent the patient’s participation in the study as well as diagnosed mental incapacity. Prior to enrolment, all participants signed an informed consent form. All clinical investigations were conducted considering the Declaration of Helsinki. The Research Ethics Committee of Cádiz approved the study protocol on 26 October 2017 (registration number 84.17).

### 2.2. Study Design

The experiments were carried out at the UHPM, where it was possible to observe the participants closely, make precise recommendations and conduct them under medical and technical supervision. The tests were held under comfortable temperature and humidity conditions.

**Figure 1:**
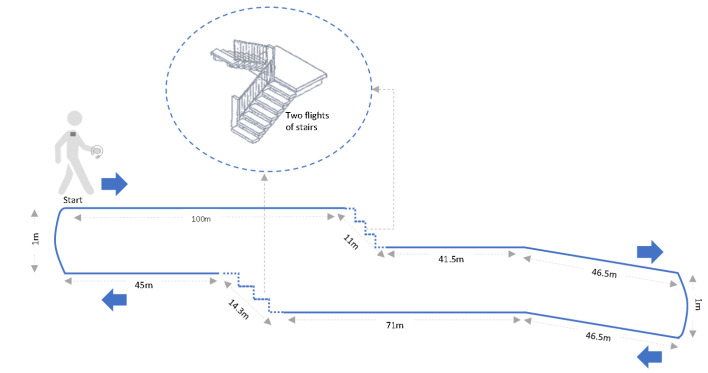
Route design of the 20-minute walk circuit.

For the 6MWT, a straight and flat internal corridor, 30 m long, was used. For the 20MWC, a closed-circuit was designed including representative sections of the activities carried out in daily free-living conditions: walking on the flat, a sloping area and a section of stairs going down and up (Figure 1). The endpoints were signposted on both routes, and chairs were arranged throughout both circuits so that the patient could rest if he or she found it convenient.

Basic guidelines for the implementation of both tests were given to patients [16]. In the case of the 6MWT, participants were asked to walk as far as possible while in the 20MWC, participants were told that the intensity of walking should be the usual one in their daily routine. The duration of 20MWC was set at 20 minute. Both tests were conducted by study researchers, who walked behind the patient and made notes on different situations of interest according to the data collection protocol.

The 6MWT was performed by patients at the oxygen flow they were titrated for exertional situations. In the case of the 20MWC, patients were indicated to modify the oxygen flow or pulse depending on whether they were at rest or on exertion, according to the previous titration process personalized for the patient. All participants were prescribed for exertional oxygen therapy in pulse mode. Oxygen devices from several manufacturers (Inogen One by Inogen, Goleta, CA, USA; SimplyGo by Philips Respironics, Murrysville, PA, USA; Invacare XPO_2_ by Invacare Corporation, Auckland, NZ; and EasyPulse POC by Precision Medical, Northampton, PA, USA) that counted with national approval were used by patients.

Participants followed the designed study protocol. First, an interview was conducted to inform of the study objectives, and data were collected under the patient’s baseline conditions. Subsequently, the 6MWT was performed, under the conditions described above. After a 30-minute rest period, the 20MWC was started. Immediately after each test, the variables of interest that required the direct participation of the patient were recorded (i.e., blood pressure and measurements of dyspnoea and leg fatigue).

Patients used their own portable oxygen therapy equipment for both tests. Following the last run, after a 30-minute resting period, and when the patient returned to a basal, stable condition with no symptoms or signs of alarm, the test was concluded. During each test, the stop and start times for each activity were recorded and a label was assigned to each period. Besides, physiological and subjective measures, derived from both tests, were collected.

### 2.3. Physiological Variables and Performance during Tests

**Figure 2:**
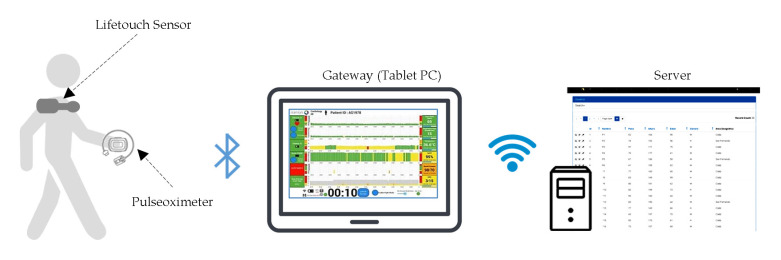
Functional architecture of the monitoring system architecture used in the effort tests.

Patients were monitored using Lifetouch (by Isansys Lifecare Ltd., Oxfordshire, UK) and Nonin WristOx 3150 (by Nonin Medical Inc., Plymouth, MN, USA) sensors. Figure 2 illustrates the functional architecture of the monitoring system used in the tests. The Lifetouch wireless portable biosensor is a wireless patch with two ECG electrodes that is placed on the left front side of the chest. This sensor records the heart rate (HR), the electrocardiographic wave and allows to see in real-time alterations in the trace or heart rhythm. The Nonin 3150 WristOx sensor is a wearable pulse oximeter that provides a measurement of arterial oxygen saturation (SpO_2_) using a standard finger probe.

Data from both sensors were stored in real-time and transmitted using Bluetooth Low Energy to a patient gateway (modified Samsung tablet). In the study, this gateway was connected via Wi-Fi to a server hosted behind the Hospital’s firewall, where there was an electronic patient record to which the information collected during each test was linked.

SpO_2_ was recorded in baseline condition, every second during the test, and at the end of the test. The percentage of time spent with SpO_2_ above 95% and below 90%, 88%, 85%, 80%, 75% and 70% was determined. An oxygen desaturation event during the test was defined as a drop in SpO_2_ ≥ 4% for at least 10 s [29].

Likewise, the heart rate and breathing rate were measured, in the baseline situation, during the test and at the end of each circuit. Blood pressure was measured in the basal state and at the end of each test. Concerning performance during the test, the following parameters were counted: the meters walked; the number of stops during each test, the time associated with each of these pauses and the number of these resting episodes in which the patient needed to be seated.

For the estimation of energy expenditure, the non-linear mathematical model recently proposed for 6MWT by Szczegielniak et al. [30] was used, which provides the energy cost or metabolic equivalent (MET) of the physical activity performed as a function of the distance travelled during the test and taking into account gender differences, which can be more efficient in terms of the non-linearity of the environment than the classical method of Connors and Hilling [31].

### 2.4. Subjective Measurements

Habitual breathlessness was assessed using the modified Medical Research Council (mMRC) scale [32]. Dyspnoea measurements before and after the tests were collected using the modified Borg scale [33]. The perception of leg fatigue was assessed at the end of each circuit, at rest and using the same Borg scale.

### 2.5. Statistical Analysis

The normality of the distribution of the variables was verified by the Shapiro Wilk test. The descriptive analysis of each continuous variable was expressed as mean ± standard deviations and 95% of confidence interval, or median and interquartile range according to the normality. Categorical variables were described as number and percentage. The comparison between the results of the 6MWT and 20MWC tests was made using the Student t-test (parametric) or the Wilcoxon test (non-parametric) as a function of normality. The correlation between the two tests and between anthropometric data and test results was calculated using the Pearson (parametric) and Spearman (non-parametric) correlation coefficient. In the case of binary variables, the correlation was estimated using the Mathew correlation coefficient. The statistical analysis was carried out using Matlab (Mathworks Inc., Natick, MA, USA) mathematical software. A value of *p* < 0.05 was considered significant.

## 3. Results

### 3.1. Participants

The study group was heterogeneous and consisted of fourteen subjects with COPD and three with diffuse interstitial lung disease (DILD). The average age of the study group was over 75 years old and the participants had been prescribed with LTOT on average 23 months before the study. Oxygen dosage had been titrated by the 6MWT. They were all users of portable O_2_ concentrators for an average of 21 months. The clinical and functional characteristics of the patients are shown in Table 1.

### 3.2. Distance

On average, the distance walked by patients during the 6MWT (6MWD) was 287 ± 94 m and 577 ± 285 m during the 20MWC (20MWD). The proportion of subjects with a distance covered shorter than 350m in 6MWT was 76% (Table 2).

A good correlation was found between 6MWD and 20MWD (r = 0.53; *p* = 0.027). To establish the abnormality, theoretical values were estimated using the equation of Casanova et al. [34] whose data are more closely adjusted to the Spanish population than other frequently used equations [18,35,36]. According to the personalized values, in 41% of the participants, 6MWD was lower than the theoretical threshold established by the above equation.

### 3.3. Physiological Response

Table 2 summarizes the resulting parameters related to physiological and performance variables during the 6MWT and the 20MWC.

**Figure 3:**
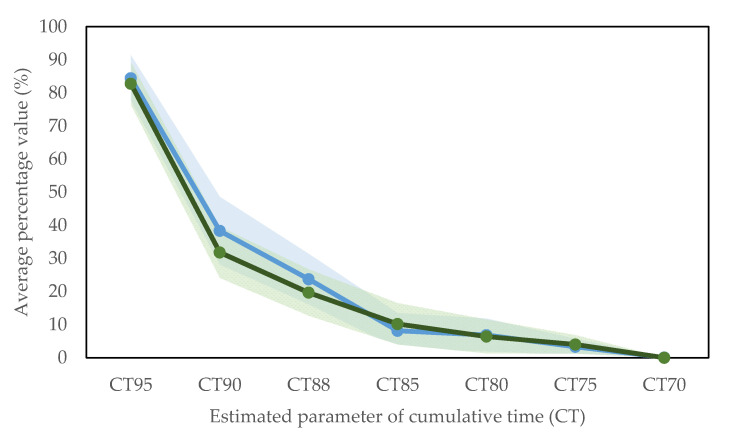
Mean and standard error bands for cumulative time percentages during the two walking tests for SpO_2_ < 95% (CT95), SpO_2_ < 90% (CT90), SpO_2_ < 88% (CT88), SpO_2_ < 85% (CT85), SpO_2_ < 80% (CT80), SpO_2_ < 75% (CT75), and SpO_2_ < 70% (CT70). The blue coloured line represents the average values collected in the 6-minute walking test (6MWT) and the green coloured line represents the values measured in the 20-minute walking circuit (20MWC).

Figure 3 represents various percentages of cumulative time calculated for both tests. The most pronounced difference is observed for CT90. The average value in the 6MWT was 6.5 percentage points higher than the average value estimated in the CM20M.

Although the mean CT90 in the 20MWC was lower than its equivalent in the 6MWT for 41% of the patients (7 out of 17), differences were not statistically significant. Furthermore, considering only the patients who presented a CT90 > 0 in both tests, we found that the CT90 was lower in the 20MWC than in the 6MWT for 58% of the patients.

Mean CT95 during the 20MWC was 82.7%, which indicates that, on average the subjects spent 17.3% of the time with a SpO_2_ ≥ 95%. As detailed in Table 2, no significant statistical differences were found between CT95 in both tests. The number of patients who presented episodes with SpO_2_ > 95% was significantly greater during the 20MWC (n = 10) than during the 6MWT (n = 4, *p* = 0.0312).

Regarding the highest blood oxygen saturation, patients that presented a SpO_2_ > 95% at some stage of the test were 10 in the 20MWC and 4 in the 6MWT.

The estimated average energy expenditure during the 6MWT was 3.1 ± 0.9 METs. In the 6MWT, the mean number of rest stops made by patients, the percentage of time stopped, and the number of seated rest stops during the experiments were 0.7 ± 0.8, 14.6 ± 23.5% and 0.5 ± 0.9 respectively. In the case of the 20MWC, these figures were 5.1 ± 1.9, 54.4 ± 32.0%, and 2.7 ± 1.1, respectively.

### 3.4. Subjective Measurements

Variations in dyspnoea and leg fatigue were similar in both tests, as detailed in Table 3.

## 4. Discussion

Clinical guidelines state that LTOT is a proven treatment associated with a lower mortality rate for patients with chronic hypoxemia if administered at least 15–16 h a day [37].

In recent years, technical advances have made it possible to administer it continuously and when the patient is out of the home. To ascertain the prescription of oxygen therapy for ambulation it is requested that the patient have a decrease in oxygen saturation during exercise which can be compensated by portable oxygen administration. Home oxygen therapy using portable concentrators to facilitate ambulation is commonly designed and customized through the 6MWT. Evidence from studies with COPD patients indicates that 6MWT is an effective method of detecting exercise-induced hypoxemia during activities of daily living and establishing the oxygen flow needed to correct exertional desaturation [38].

Nevertheless, the conduct of the 6MWT has intrinsic features. It has been highlighted that patients usually tend to start walking at a much faster pace than the constant speed they achieved afterwards. It implies that short walking tests tend to measure initial effort rather than true exercise tolerance [22]. Accordingly, it has been suggested that a longer duration test allows for a better assessment of the patient’s functional capabilities [39]. Indeed, it has been observed that changes in the VO_2_max correlate better with changes in the 12-minute test than in shorter walking tests [40]. Furthermore, since the 6MWT is a self-regulated test, performance depends on external factors such as energy expenditure, the motivation generated by the clinician who accompanies the test and the patient’s intrinsic motivation [41,42].

As far as we know, no study has directly evaluated the patient’s response to a walking test that more closely represents the physical activities carried out in daily life, and while the patient is using an O_2_ supply device governed according to the indications made in the personalized titration process.

To conduct the tests, a walking circuit was designed. The circuit was initially designed to evaluate an intelligent portable concentrator that responded to changes in the subject’s physical activity and automatically updated the oxygen flow/pulses without the need for the patient intervention [43]. In this circuit, the patient was instructed to walk at his/her usual pace, as opposed to 6MWT, where the patient is instructed to walk as fast as possible without running or jogging.

From the analysis of the results gathered, some significant findings have emerged. Concerning the distance walked, 6MWD and 20MWD were well correlated. Regarding the oxygen saturation features, differences were found in CT90 and CT95. Average CT90 estimated was 6.5% greater in the 6MWT than in the 20MWC. In 41% of the patients, the average CT90 during the 20MWC was lower than during the 6MWT. For the rest of patients in which this situation did not comply, a possible underlying cause might lie in the rest stops that these patients made during the tests.

Additionally, it is noteworthy that the average CT95 estimated from the 20MWT was 82.7%, which implies that the patients spent a considerable amount of time with an oxygen saturation over 95%. The number of patients with episodes with SpO2 > 95% was significantly greater in the 20MWC compared to the 6MWT. While the purpose of oxygen therapy is to avoid hypoxemia, the risk associated with induced hyperoxia is often overlooked [13]. This risk is present if the dose of oxygen needed at rest or during the performing of daily physical activities is not adequately titrated.

Although the lack of a consensual definition of hyperoxia, some authors have defined 96% as a safe upper SpO_2_ limit. A sharp increase in the prevalence of hyperoxia has been found when SpO_2_ rises above 95% in ICU patients [44]. Consequently, data revealed that 6MWT could lead to excessive O_2_ titration for certain activities of daily life.

Although most of the described risks of hyperoxia are associated with acute patients, O’Donnell et al. [45] reported in their study that 9 out of 20 patients with stable COPD increased PaCO_2_ to 60% FiO_2_ after 10 min of hyperoxia. Despite the levels of oxygen delivery that can be provided using portable devices are not usually very high, they may generate SpO_2_ levels > 95%, which maintained for long time intervals could have deleterious effects in some patients such as CO_2_ retainers.

It must be assumed that the majority of daily activities performed by patients with COPD is light or sedentary (METs < 3) with more than 40% of the time under sedentary behaviour. However, the average energy expenditure calculated during the 6MWT was greater than three METs. Again, an overestimation by the 6MWT of the intensity of exercise performed by these patients is observed when compared to the actual physical activity characterized in recent studies.

In this regard, the 20MWC might be an alternative to the 6MWT to better estimate the exercise capacity in patients with respiratory failure and it is also more suitable to set the oxygen therapy. Despite the 20MWC does not proportionally represent daily life activities, the combination of flat with stair sections may serve as an approximation to the scenarios of everyday life, presenting the patient with different biomechanical and metabolic requirements [46].

The supply of excess oxygen to the patient has implications that are beyond the straightforward consequence on the patient’s safety. It affects the autonomy of modern portable oxygen concentrators. This cannot be overlooked, given that one of the main barriers to the acceptance of portable oxygen devices by patients refers to the low autonomy device [47]. The supply of a higher oxygen flow rate than necessary negatively affects the device-autonomy, causing it to decrease. The potential enhancement in battery performance would also support the promotion of physical activity outside the home.

In this study, the efficiency of the personalization of LTOT has been discussed and a new 20-minute test evaluated. Our study has limitations. Firstly, the study of the learning effect was not able to be completed because it requires the double realization of each circuit. This is particularly challenging given the physical demands of the experiments. Secondly, the limited sample size poses a constraint on the generalization of the findings. Due to the small sample size, findings should be interpreted cautiously. A larger group with additional subjects would help to contrast the generalization of the results obtained. Finally, further studies are needed including the arterial blood gas analysis before and after exercise, which could add relevant information that might lead to define specific patient profiles.

In conclusion, we found that, in our clinical population, mean CT90 was higher in the 6-minute walking test than in a longer test designed ad-hoc to reproduce daily physical activities more truthfully. A significant percentage of patients presented a CT90 in the 6MWT higher than in the 20MWC. In addition, a significant sample of patients was found to have episodes of oversaturation (> 95%) in 20MWC. Finally, the estimated caloric expenditure for the 6MWT was higher than the one published in studies that assess the actual intensity of the physical activity of patients in a domiciliary environment.

The results indicate that in a significant proportion of patients, the 6MWT might not accurately predict their oxygen needs at a domiciliary environment. Therefore, the titration of the portable O_2_ therapy could not be optimal in these cases, with the detrimental impact on the patient’s health (hyperoxia episodes), the autonomy of the oxygen device, and the decrease of time out of the home. Therefore, we consider that more research is needed on the home monitoring of oxygen saturation of patients under LTOT to assess whether the oxygen therapy received at home meets the actual and personalized needs of each patient.

## Figures and Tables

**Table 1:** Clinical and functional characteristics in the study group.

Feature	Mean ± SD	Range
Male/Female	15/2	-
Age, years	67.1 ± 6.3	54–80
BMI, kg/m^2^	26.3 ± 4.0	18.0–33.1
Moths under LTOT	23.1 ± 19.5	3–72
FEV1, %	42.2 ± 19.7	23.0–94.0
FEV1/FVC	0.5 ± 0.2	0.2–0.8
mMRC	2.5 ± 0.6	1.0–3.0
Dyspnoea, Borg	0.2 ± 0.5	0.0–2.0
Time out of home, hours	3.1 ± 1.8	1.0–8.0

SD: standard deviation; BMI: body mass index; LTOT: Long term oxygen therapy; FEV1: forced expiratory volume in the first second; FVC: forced vital capacity; mMRC: modified dyspnoea scale (modified Medical Research Council).

**Table 2:** Physiological and performance variables during the 6-minute walk test (6MWT) and the 20-minute walk circuit (20MWC). Values are expressed as mean ± standard deviation, with 95% confidence interval in brackets. Categorical variables were described as number and percentage. CT90: cumulative time with SpO_2_ < 90%; CT95: cumulative time with SpO_2_ < 95%; SpO_2_: arterial oxygen saturation measured by pulse oximetry; HR: heart rate; bpm: beats per minute.

Variable	6MWT	20MWC	*p*-Value *	Correlation Coefficient with CI95%
Distance walked (m)	287 ± 94 [238.7–335.4]	577 ± 285 [431.4–724.2]	0.0002	r = 0.53 [0.07–0.80], *p* = 0.027
Rest stops	0.7 ± 0.8 [0.3–1.1]	5.1 ± 1.9 [4.1–6.0]	< 0.0001	r = 0.45 [−0.04–0.76], *p* = 0.071
Seated rest stops	0.5 ± 0.9 [0.0–0.9]	2.7 ± 1.1 [2.1–3.3]	< 0.0001	r = 0.66 [0.27–0.87], *p* = 0.003
CT90, %	38.4 ± 42.3 [16.7–60.1]	31.8 ± 32.1 [15.3–48.3]	0.2783	r = 0.82 [0.56–0.93], *p* < 0.001
CT95, %	84.4 ± 28.5 [69.8–99.1]	82.7 ± 26.7 [69.0–96.5]	0.7530	r = 0.70 [0.33–0.88], *p* = 0.002
Patients with SpO_2_ > 95%	4 (23)	10 (59)	0.0312	ϕ = 0.46 [−0.02–0.77] *, *p* = 0.001
Mean SpO_2_, %	89.8 ± 4.2 [87.6–91.9]	90.1 ± 5.2 [87.5–92.8]	0.5961	r = 0.85 [0.63–0.94], *p* < 0.0001
Mean HR, bpm	103.1 ± 12.9 [96.5–109.7]	102.6 ± 12.9 [95.9–109.2]	0.8166	r = 0.72 [0.36–0.89], *p* = 0.001
Max HR, bpm	111.0 ± 12.3 [104.7–117.3]	121.0 ± 21.5 [109.9–132.0]	0.0303	r = 0.59 [0.16–0.84], *p* = 0.012
Nadir SpO_2_, %	86.6 ± 6.8 [83.1–90.1]	85.5 ± 6.5 [82.1–88.8]	0.1657	r =0.87 [0.68–0.95], *p* < 0.0001

* Mathew correlation coefficient (ϕ).

**Table 3:** Perceived differences in dyspnoea and leg fatigue measured at the beginning and end of each test. Δ Dyspnoea = Dyspnoea after the test − Dyspnoea before the test; Δ Leg Fatigue = Leg Fatigue after the test − Leg Fatigue before the test.

	6MWT	20MWC	*p*-Value	Correlation Coefficient with CI95%
**Δ Dyspnoea**	2.2 ± 2.1 [1.1–3.3]	4.2 ± 2.2 [3.0–5.3]	0.0092	r = 0.22 [−0.29–0.63], *p* = 0.4006
**Δ Leg Fatigue**	0.4 ± 0.9 [−0.1–0.9]	1.2 ± 1.8 [0.3–2.2]	0.1250	r = 0.44 [−0.05–0.78], *p* = 0.0739
